# Bilateral Primary Adrenal Lymphoma Presenting with Adrenal Insufficiency

**DOI:** 10.1155/2012/638298

**Published:** 2012-09-04

**Authors:** Jakob Holm, Leif Breum, Katrine Stenfeldt, Mette Friberg Hitz

**Affiliations:** ^1^Department of Medicine, Endocrine Unit, Koege Hospital, University of Copenhagen, 4600 Koege, Denmark; ^2^Department of Pathology, Roskilde Hospital, University of Copenhagen, 4000 Roskilde, Denmark

## Abstract

Lymphoma may occasionally involve the adrenal glands, but primary adrenal lymphoma (PAL) is very rare and only few cases have been reported. We present a case of a 60-year-old, otherwise healthy, woman, with bilateral PAL presenting with adrenal insufficiency. The patient responded initially upon administration of large doses of intravenously hydrocortisone with total remission of symptoms. An abdominal computerized tomography scan demonstrated bilateral adrenal lesions but did not demonstrate any other pathology. Since metastatic malignant disease was suspected a positron-emission-tomography scan was performed only showing significant uptake in the adrenal glands. Endocrine evaluation did not reveal abnormal function of any hormonal system and the patient was scheduled for bilateral adrenalectomy. However the clinical condition deteriorated rapidly and the patient was readmitted to hospital before surgery was performed. A new computerized tomography scan showed rapid progression of disease with further enlargement of the adrenal masses and both pulmonary and hepatic metastasis. Needle biopsy was performed but the patient refused further treatment and died before a diagnosis was obtained. The immuneohistochemical diagnosis was large B-cell lymphoma. This case should remind clinicians that PAL may be a cause of bilateral adrenal incidentaloma especially if the patient presents with adrenal insufficiency.

## 1. Introduction

Primary adrenal lymphoma (PAL) is rare whereas secondary involvement of the adrenal glands in nodal non-Hodgkin lymphoma occurs more often and is present in approximately 25% of cases [1].

Primary extranodal lymphoma occurs in 1/3 of patients but primary involvement of the endocrine system is not frequent (3%) and most often involves the thyroid gland [2].

The rare cases of PAL may present with bilateral (bPAL) involvement and is then more often histologically of diffuse large B-cell type. Immunodysfunction, as observed with human immunodeficiency virus infection or autoimmunity, may predispose to the disease but is not obligate [3].

Adrenal insufficiency may be the primary symptom of presentation, especially with bilateral involvement as in bPAL [4].

A high degree of suspicion is important in order to obtain a diagnosis quickly and initiate treatment since prognosis is poor.

## 2. Case Presentation

A 60-year-old woman previously diagnosed with systemic lupus erythematosus, but without symptoms of disease or need of treatment for the last 10 years, was admitted to our hospital due to a month's history of nausea, vomiting, fatigue, and fever. According to the patient an unintended weight loss of 5 kilograms had occurred. The patient took no prescribed medications and had no other medical history.

Primary physical evaluation revealed a normal blood pressure and temperature. Clinical examination was unremarkable especially no lymphadenopathy, hepatosplenomegaly, or skin pigmentation was observed.

Laboratory examination showed severe hyponatremia with a sodium level of 106 mmol/L (137–145 mmol/L) and a potassium level of 4.5 mmol/L (3.6–5.0 mmol/L). Liver parameters were slightly affected, coagulation factor II, VII, X was 0.55 (0.7–1.3), and lactate dehydrogenase was 292 U/L (105–205 U/L). C-reactive protein was 35 mg/mL (0.2–8 mg/mL) and the patient had slight thrombocytopenia 92 × 10^9^/L (145–390 × 10^9^/L). Blood sugar was normal. 

 Due to the symptoms and the severe hyponatremia, adrenal insufficiency was suspected and an ACTH stimulation test was performed. The test demonstrated an insufficient response with an increase from baseline plasma cortisol of 222 pmol/L (190–600 pmol/L) to 239 pmol/L (>500 pmol/L) after 30 minutes and an elevated plasma ACTH of 75 pmol/L (2–11 pmol/L), indicating a primary adrenal insufficiency.

The patient was treated with high doses of intravenous hydrocortisone and rehydrated with sodium chloride infusion resulting in complete remission of symptoms and normalization of biochemistry.

Further biochemical evaluation showed no antibodies against the adrenal cortex. Plasma renin was 46 miU (8.8–36 miU) and plasma aldosterone was 38 pmol/L (38–490 pmol/L). 

Urine sodium was subnormal <20 mmol/L (50–150 mmol/L) and urine osmolarity was also low as expected 286 mmol/kg (>800 mmol/kg).

A Quantiferon test ruled out Mycobacterium tuberculosis infection and phaechromocytoma was ruled out as well by the measurement of plasma metanephrine of 79 ng/L (0–170 ng/L) and normetanephrine <20 ng/L (0–72 ng/L). 

**Figure 1:**
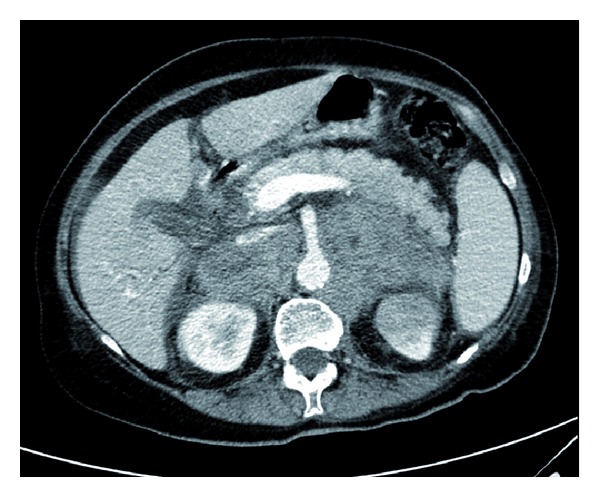
Abdominal CT scan showing large bilateral adrenal masses of homogenous appearance. No other pathology was demonstrated.

A contrast enhanced computerized tomography (CT) scan of the chest and abdomen revealed solid bilateral adrenal masses of 4 cm × 7 cm in the left adrenal gland and 6 × 3 cm in the right adrenal gland (Figure 1). Masses were homogenous and Hounsfield score was high. No other pathology was demonstrated.

Adrenal metastasis from unknown primary cancer was suspected and a positron-emission-tomography scan was performed. The scan demonstrated significant uptake in the adrenal glands no primary tumor was found and no enlarged lymph nodes were detected.

The patient preferred diagnostic adrenalectomy over needle biopsy and was referred to the Department of Urology in order to perform diagnostic bilateral adrenalectomy, but the clinical condition deteriorated and the patient was readmitted to our hospital before the surgical procedure could be performed. A new enhanced CT scan showed rapid progression of the adrenal masses as well as pulmonary and hepatic metastases. In order to be able to obtain a diagnosis and possibly treat the patient, a needle biopsy was performed. Initially a histological analysis was done on a frozen section in order to be able to offer some empiric treatment to the patient, but tentative diagnosis was too unspecific to offer chemotherapy. 

**Figure 2:**
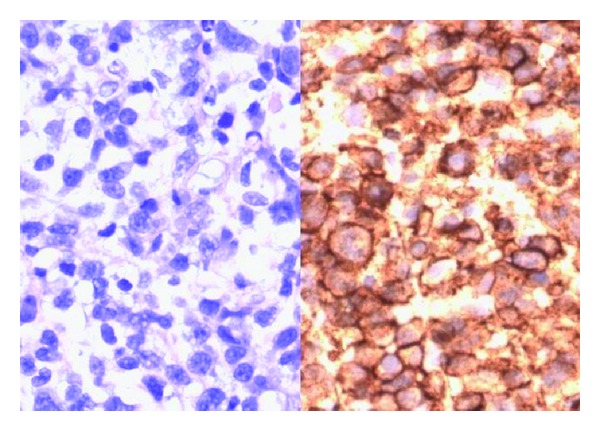
H&E stain and immunostaining for CD20 (×400) showing large dysplastic CD20 positive B-lymphocytes.

Subsequently the patient refused further treatment and died before a final pathological diagnosis was known. Pathological examination showed biopsy consisting of solid tumor tissue only, with no organ specific structures. The tumor cells were large with abundant basophilic cytoplasm and irregular nuclei, many with a nucleolus. The tumor cells expressed CD20, CD45, CD79a, Bcl-2, vimentin, CD38, and Kappa the diagnosis was thereby established as diffuse Large B-cell lymphoma (Figure 2). The proliferative fraction detected by Ki-67 was high, 60%. The tumor cells stained negative in S-100, AE1/AE3, CD3, CD5, CD10, CD30, and lambda.

## 3. Discussion

An increasing number of adenomas in the adrenal glands are now diagnosed due to an increasing number of abdominal CT scans being performed. Incidentalomas are defined as tumors in the adrenal glands found by accident when performing diagnostic imaging for nonadrenal symptoms [5].

Less than 5% of the incidentalomas are malignant and only 1 in 7 shows excessive production of any adrenal hormone (cortisone, catecholamine,or aldosterone). Incidentalomas are often unilateral [6].

Diagnostic imaging with nonenhanced CT scans is used primarily to characterize the tumor. Low Hounsfield score, homogeneous morphology, and small size indicate that tumor is benign. Further evaluation with contrast enhanced CT of the abdomen and calculation of percentage wash-out can be used in need for further evaluation. Fine needle biopsy or adrenalectomy is used when a histological diagnosis is needed. If the tumor is hormone producing or if the tumor is large adrenalectomy is performed [7]. The patient presented here had symptoms of adrenal insufficiency and cannot traditionally be characterized as an incidentaloma. It is important to have the diagnostic possibility of bPAL present when evaluating patients with adrenal mass, especially if bilateral masses are present. 

Lymphoma may spread to any part of the body and involvement of the adrenal glands in malignant lymphomas is reported in 25% of autopsies. 

Bilateral adrenal tumors often represent metastasis. Primary lung or stomach tumors are the cause in 50% of the cases and metastasis from lung, breast, stomach and lymphoma is the most common course of adrenal metastasis giving rise to adrenal insufficiency. PAL on its own is an extremely rare disease entity and less than 100 cases have been reported in the last 40 years [8].

PAL is rare and often presents with bilateral tumor masses (70%) and can results in adrenal insufficiency. Survival time is short and a high degree of suspicion is needed in order to obtain a quick diagnosis. Advancing age, tumor size, level of LDHs and the presence of adrenal insufficiency are poor prognostic signs [9].

Most patients with PAL have a limited time of survival. Complete remission of disease after initiation of chemotherapy have been described in a few patients, with a followup of 12 months in one patient without remission of disease and a followup of 7 years in another patient with no signs of remission [10–12].

## 4. Conclusion

Primary adrenal lymphoma is rare but most often present with bilateral tumors and symptoms of adrenal insufficiency. A high degree of suspicion is needed in order to obtain a quick diagnosis since prognosis is extremely poor.
